# Bi/mZVI Combined with Citric Acid and Sodium Citrate to Mineralize Multiple Sulfa Antibiotics: Performance and Mechanism

**DOI:** 10.3390/antibiotics11010051

**Published:** 2022-01-01

**Authors:** Xiaoming Su, Hao Lv, Jianyu Gong, Man Zhou

**Affiliations:** 1 School of Environmental Science and Engineering, Huazhong University of Science and Technology, Wuhan 430074, China; m202073955@hust.edu.cn (X.S.); m202174048@hust.edu.cn (H.L.); 2 Hubei Electromechanical Research Institute Co., Ltd., Wuhan 430070, China

**Keywords:** sulfanilamide drugs, Bi/mZVI, antibiotic, oxidative degradation

## Abstract

The oxidative mineralization of sulfanilamide drugs (SAs) using micro-size zero-valent iron (mZVI) cooperated with a citric acid buffer solution was evaluated. In this study SM2, SMX, and SD could be removed at 66%, 89%, and 83%, respectively, in a 0.5% Bi/mZVI+CA+NaCA system within 2 h. Based on our analysis, the produced ·OH could be ascribed from the complexation between citrate iron (Fe(II)[Cit]^−^) and the generated H_2_O_2_ resulting from the activation of O_2_ on the mZVI surface in the Bi/mZVI+CA+NaCA system, further inducing the mineralization of antibiotics. The related possible degradation pathways were proposed. Two similar degradation pathways of SM2, SMX, and SD in the mixed liquid, including hydroxylation and SO_2_ extrusion, were solved. Meanwhile, there was an additional proposed degradation pathway for SMX to be degraded more effectively, as reflected in the opening of the N-O bond on the benzene ring. Therefore, this work provides an experimental basis and theoretical support for the efficient treatment of antibiotic wastewater in real industry by using an iron-based method.

## 1. Introduction

Sulfanilamide drugs (SAs) are the most common type of antibiotics to treat infectious diseases in humans and animals. Although the emission concentration of SAs is not high, they will gradually accumulate in aqueous solutions, due to their long-term structural stability, causing irreversible effects, such as carcinogenesis and gene mutation induction [1]. 

Nowadays, many studies have focused on the degradation of SAs by advanced oxidation processes (AOPs), including photocatalytic oxidation, electro-catalysis, persulfate oxidation, and Fenton oxidation [2,3,4,5]. However, each of these technologies has its own shortcomings [6,7], such as the photo-catalysis or electro-catalysis technology’s need for expensive equipment and hard installation. In addition, the persulfate oxidation or Fenton reaction requires the addition of oxidants, which restricts their development due to their high costs.

Zero-valent iron (ZVI), which is one of the most abundant metals in the earth’s crust, was widely used for the removal of heavy metal(loid)s [8,9,10] and organic chlorinated pollutants [11,12,13], due to its strong reduction ability, low price, ease to obtain, and environmental friendliness. However, there are some problems when using ZVI to degrade organic pollutants. The reducibility of ZVI may lead to longer time, lower degradation efficiency, and low mineralization [14]. Therefore, finding some new strategies to improve the reactivity of ZVI is the key point to enhancing the degradation of organic pollutants. Many studies have found that the activation of O_2_ to form H_2_O_2_ is an effective method [15,16,17]. The way of activation of O_2_ to form H_2_O_2_ and then generate reactive oxygen species (ROS) such as ·OH and ·O_2_^−^, which can effectively mineralize organic pollutants and reduce the production of higher toxic by-products. Generally, the processes of activation of O_2_ on iron and further degradation of organic contaminant are described as follows (Equations (1)–(5)).

Fe^0^ + O_2_ + 2H^+^ → Fe^3+^ + H_2_O_2_ (1)

Fe^2+^ + O_2_ → Fe^3+^ + ·O_2_^−^ (2)

Fe^2+^ + 2H^+^ + ·O_2_^−^ → Fe^3+^ + H_2_O_2_ (3)

Fe^2+^ + H_2_O → Fe^3+^ + ·OH + ·O_2_^−^ (4)

Fe^3+^ + H_2_O_2_ → Fe^2+^ + H^+^ + ·OH_2_ (5)

The addition of chelating agents can remarkably enhance the ability to activate O_2_. Some common ligands, such as EDTA [18], oxalic acid [19], and humic acid [20] can form complexes with Fe(II) or Fe(III) to promote the degradation of contaminants. In addition, the formed iron complexes can be adsorbed on the surface of ZVI to hinder the deposition of iron oxide compounds on the surface, thereby alleviating the passivation of ZVI [21]. At the same time, the iron complex could slow down the dissolution of iron and prolong the working life of the ZVI. However, choosing a suitable ligand is also particularly critical. As a ubiquitous substance in nature, citric acid (CA) has the characteristics of low cost, green, and environmental friendliness. Therefore, it is a ligand with great application prospects. The general mechanism is as follows (Equations (6) and (7)).

Fe^2+^ + Cit_3_^−^ → Fe(II)(Cit) (6)

Fe(II)(Cit) + H_2_O_2_ → Fe(III)(Cit) + OH^−^ + ·OH (7)

In order to further promote the degradation of pollutants, some transition metals or inert metals can be used to modify ZVI, and these metals include Cu [22], Ni [23], Ag [24], and so on. Among the various metals, bismuth (Bi) has attracted considerable interest due to its low cost and environmental friendliness [25]. The introduction of bismuth (Bi) onto the mZVI surface can reduce the activation energy of the reaction system to enhance the ability to oxidize pollutants [26]. Therefore, the strategy of Bi-modified zero-valent iron is feasible.

In our previous study [27,28], the nano zero-valent iron cooperated with citric acid (and sodium citrate) was utilized to degrade organic pollutants, and the mechanism of the reaction was further explored as well. However, compared with nano zero-valent iron, the micro-size zero-valent iron (mZVI) was dozens of times lower than nano zero-valent iron. Therefore, in our work, we used the method of sodium borohydride reduction to prepare bismuth modified Bi/mZVI, then the oxidative activity of Bi/mZVI in citric acid and sodium citrate was evaluated by the removal of sulfonamide antibiotics. The dissolved oxygen (DO) and the chemical oxygen demand (COD) in the reaction process were measured. The possible degradation pathways of sulfamethazine (SM2), sulfamethoxazole (SMX), and sulfadiazine (SD) in mixed liquids were proposed.

## 2. Results and Discussion

### 2.1. Characterization

**Figure 1:**
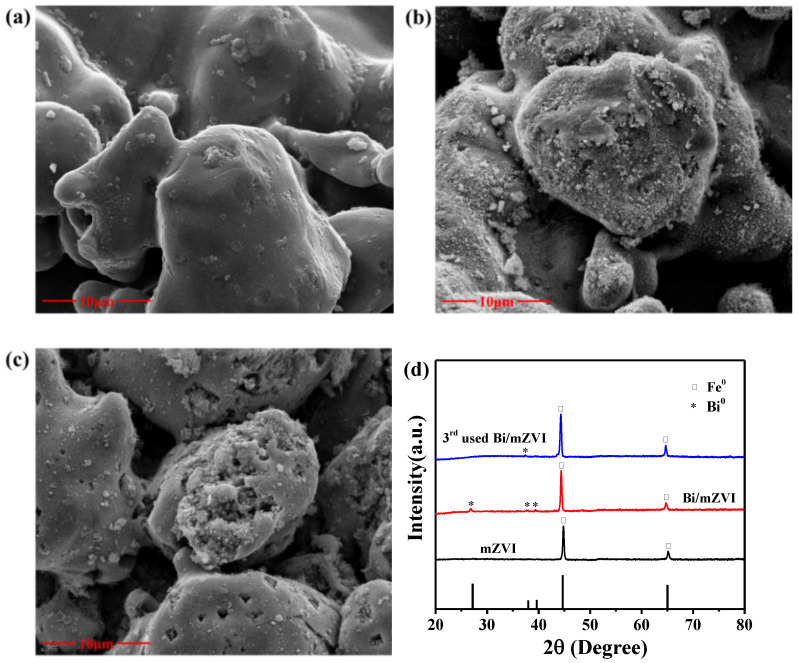
The SEM of (**a**) mZVI, (**b**) Bi/mZVI, and (**c**) 3rd used Bi/mZVI; (**d**) The XRD of different samples.

The surface morphology of the sample was characterized by SEM, as shown in Figure 1. SEM shows that mZVI (Figure 1a) is a relatively smooth particle with a small number of impurities on the surface, and Bi/mZVI (Figure 1b) is significantly rougher compared with mZVI. The reason for this phenomenon may be the reduction of the ferrite shell on the surface of mZVI, and the formation of Bi^0^ on the surface of mZVI. The SEM image of 3rd reused Bi/mZVI sample (3rd used Bi/mZVI) was shown in Figure 1c. The Bi/mZVI surface after used is still rough, which was consistent with the morphology of Bi/mZVI that has not been used. 

The crystal structures of these particles were evaluated by XRD. Figure 1d illustrated the XRD patterns of mZVI, Bi/mZVI, and 3rd used Bi/mZVI. Fe^0^ peak (2θ = 44.7° and 65°) (JCPDS No. 96-900-0659) was significantly identified for these samples. All peaks were similar to the XRD patterns of zero-valent iron observed by Xin et al. [29]. The XRD pattern of the Bi/mZVI performed this peak, and the other peaks corresponded to the Bi-crystal phase (JCPD No. 96-500-0216). For the 3rd used Bi/mZVI particle, the iron peaks were still observed implying the excellent stability of the sample in the CA+NaCA buffer system. The crystallite size of each sample were calculated by the Scherrer’s equations. The calculation results showed that the grain sizes of mZVI, Bi/mZVI, and 3^rd^ used Bi/mZVI were 19 nm, 20 nm, and 22 nm, respectively.

**Figure 2:**
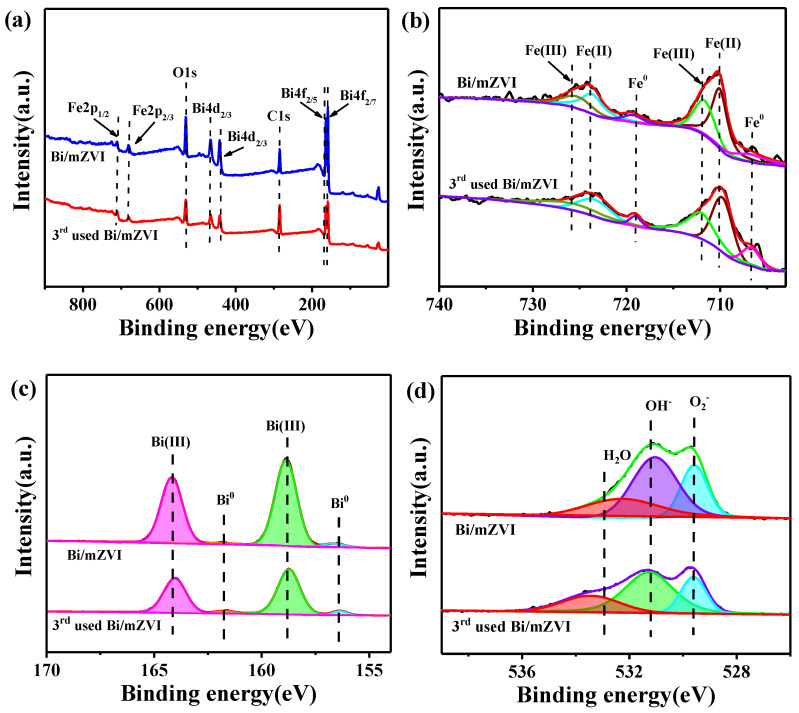
(**a**) Full XPS region of survey; high-resolution XPS spectra of (**b**) Fe 2p region (**c**) Bi 4f region and (**d**) O 1s region for the Bi/mZVI and 3rd used Bi/mZVI.

The chemical states of the samples were further investigated by XPS. Figure 2 illustrated the XPS spectra of Bi/mZVI and 3rd used Bi/mZVI. The full range of XPS spectra (Figure 2a) revealed that mZVI was composed of Fe, O, and Bi. Interestingly, as shown in Figure 2b, the iron valent had no significant change, even for the Fe^0^, which was consistent with the XRD results. Furthermore, the doped bismuth still maintained as zero valent after use, as shown in Figure 2c. However, the O peaks of the Bi/mZVI and 3rd used Bi/mZVI were changed, as shown in Figure 2d. Obviously, after continuous reaction, the peaks at 531.4 eV and 529.6 eV, which were indexed to inner oxygen coming from the iron oxide and particle surface adsorbed OH^−^, were reduced. This indicated that the iron oxide surface was firstly dissolved in an acidic condition and then the iron core was slowly exposed to the outside to involve in complexation reaction. 

### 2.2. The Oxidative Degradation of SAs

**Figure 3:**
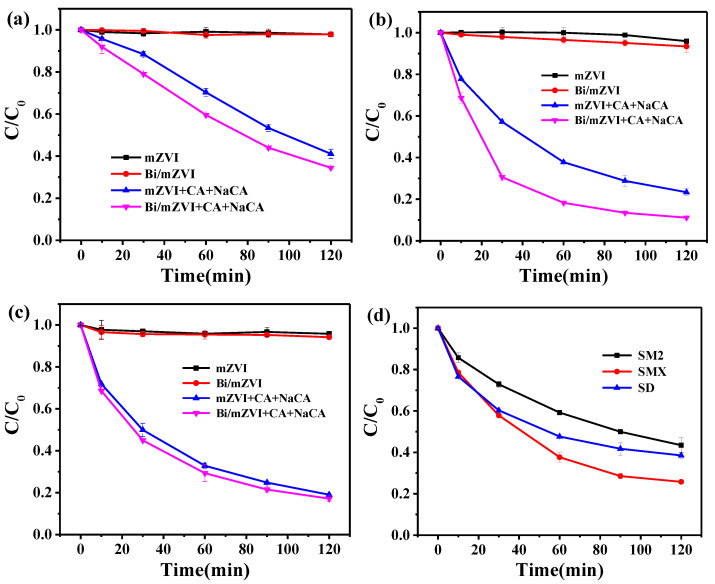

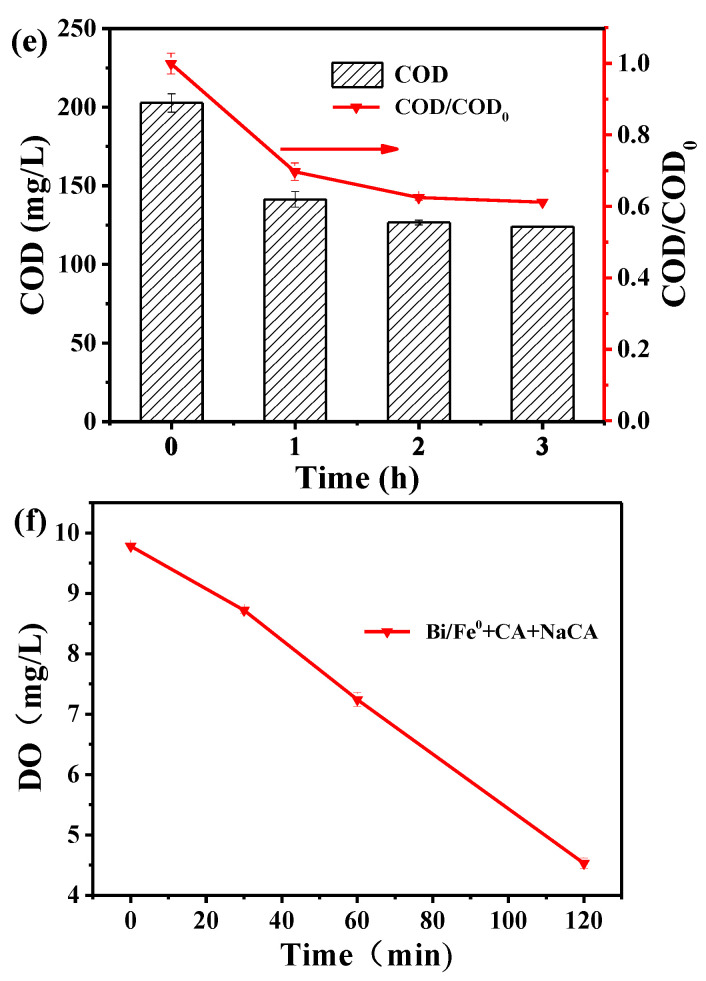
The degradation efficiencies of (**a**) SM2, (**b**) SMX, and (**c**) SD in different systems as a function of time; (**d**) The degradation efficiencies of mixed liquid in Bi/mZVI+CA+NaCA system; The variation of (**e**) COD and (**f**) DO trend of mixed liquid in Bi/mZVI+CA+NaCA system.

To optimize the content of doped Bi, the reactivity of different Bi concentration of Bi/mZVI samples toward SM2 degradation were investigated (Figure S1). Noticeably, when Bi/Fe mole ratio was 0.5%, the best degradation rate of SM2 was achieved at 65.6% in Bi/mZVI+CA+NaCA system. Therefore, the sample of 0.5%-Bi/mZVI was selected to complete all other batch experiments if without special explanation. As shown in Figure 3, SAs (SM2, SMX, SD, and the mixed liquid of these three substances) could hardly be degraded by mZVI or Bi/mZVI at the absence of CA and NaCA. However, at the presence of CA and NaCA, all cases of batch experiments could perform well removal efficiency. Figure 3a–c showed the oxidation of SM2, SMX, and SD in different systems, respectively. The removal of SM2, SMX, and SD achieved 66%, 89%, and 83% in Bi/mZVI+CA+NaCA system after 2 h of reaction, respectively. In addition, we further explored the oxidation of the three antibiotic mixed liquids in the Bi/mZVI+CA+NaCA system, as shown in Figure 3d. The degradation efficiency was 56%, 74%, and 62%, respectively for SM2, SMX, and SD, indicating the excellent oxidative activity of the Bi/mZVI+CA+NaCA system. Meanwhile, the kinetics study of SAs degradation was carried out (Figure S2). The reaction rate constants are shown in Table S1 and the degradation of SAs follows a quasi-first order kinetic process. For the degradation of single SM2 (Figure S2a), SMX (Figure S2b) and SD (Figure S2c), the reaction rate constants of Bi/mZVI+CA+NaCA system are higher than those of mZVI, Bi/mZVI and mZVI+CA+NaCA system. The results showed that Bi/mZVI+CA+NaCA system had obvious advantages in the degradation of sulfa antibiotics. Then, we investigated the kinetics process of the mixed liquid in Bi/mZVI+CA+NaCA system, and found that the reaction rate constants of SM2, SMX and SD were 6.7 × 10^−3^, 1.1 × 10^−2^ and 7.5 × 10^−3^, respectively (Figure S2d). These results indicate that CA and NaCA played an important role in the degradation of SAs. The oxidation of the three antibiotic mixed liquids in the Bi/mZVI+CA+NaCA system, as shown in Figure 3d. The oxidative degradation process of Bi/mZVI in the presence of CA and NaCA was further analyzed by chemical oxygen demand. The COD removal rate of the mixed liquid was 39% within 3 h, in addition to the COD value decreasing from 203 mg/L to 124 mg/L (Figure 3e), suggesting the feasibility of SAs degradation in Bi/mZVI+CA+NaCA system.

According to our previous study [27,28], the excitation of oxygen to produce H_2_O_2_ on the iron surface is the first process. At the same time, the citrate could complex with iron to form Fe(II)(Cit) and further oxidize the generated H_2_O_2_ to form Fe(III)(Cit) and ·OH, resulting in the oxidation of SAs. Therefore, the dissolved oxygen is important for the oxidation process. In the removal of mixed solution, the DO concentration has been exhausted from 9.7 mg/L to 4.3 mg/L at the end of the reaction, as shown in Figure 3f, implying the production of ROS. The change of pH value during the reaction process is shown in Figure S4.

**Figure 4:**
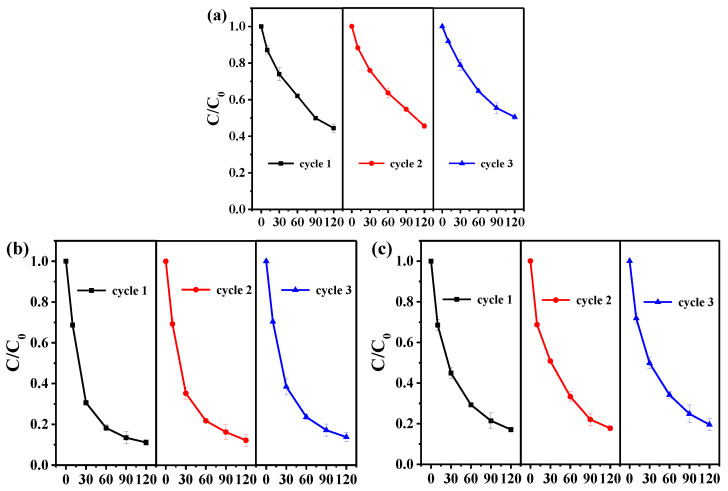
The three cycles of degradation of (**a**) SM2, (**b**) SMX, and (**c**) SD in mixed liquid by Bi/mZVI+CA+NaCA system.

To evaluate the stability of Bi/mZVI in the CA+NaCA system, three cycles of degradation of SM2 (Figure S3a), SMX (Figure S3b) and SD (Figure S3c) in Bi/mZVI+CA+NaCA system were investigated, and Bi/mZVI exhibited a strong stability in this system. Futhermore, the cyclic oxidation of the mixed antibiotic was conducted, as shown in Figure 4. Clearly, it was found that the third degradation efficiency was as good as that of the first for each compound in the mixed solution. Due to the large particle size of Bi/mZVI, the iron core had not been totally exhausted, even after the third used-in-citric-buffer-solution, so that it was still available for the next time of oxidative reaction. It was consistent with XPS analysis mentioned before.

### 2.3. The Proposed Pathway of SAs Mineralization

**Figure 5:**
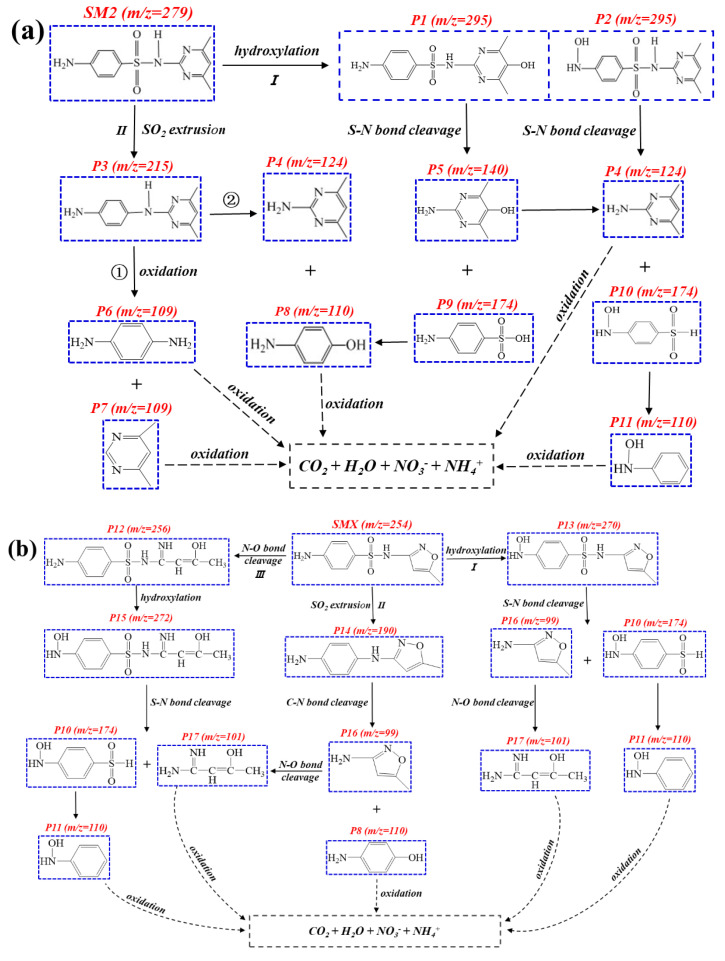

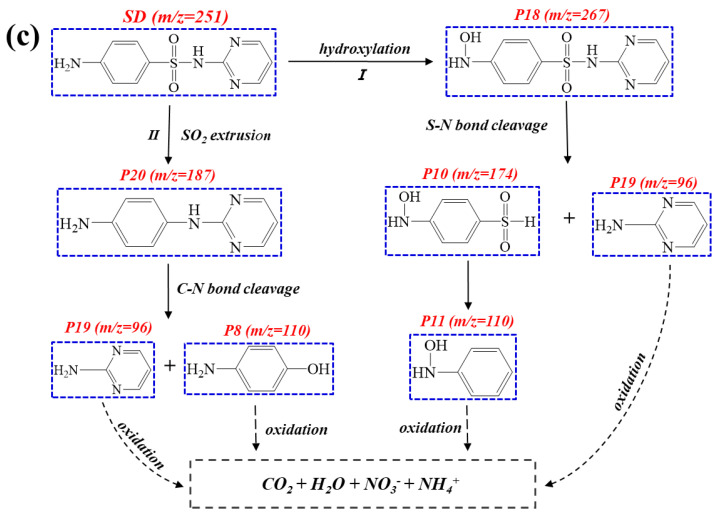
Possible degradation pathways of (**a**) SM2, (**b**) SMX, and (**c**) SD in Bi/mZVI+CA+NaCA system.

To better understand the degradation process and mechanism of SM2, SMX, and SD in Bi/mZVI+CA+NaCA system, the HR-LC-MS technology was used to identify the intermediate products of the three kinds of sulfonamide antibiotics in the mixed liquid, and 20 intermediates (Figure S5) were detected by gradient elution method (ESI^+^ mode), as listed in Table S2. The possible degradation pathways of SM2, SMX, and SD in the mixed liquid by Bi/mZVI+CA+NaCA system were proposed in Figure 5. The results of SAs degradation chromatography at different time intervals were clarified, and it was found that the chromatographic peaks of *m*/*z* = 279, *m*/*z* = 254, and *m*/*z* = 251 represented SM2, SMX, and SD, respectively (as shown in Figure S6). The degradation analysis of the mixed liquid is as follows.

Hydroxylation, oxidation, cleavage of C-N or S-N, and extrusion of SO_2_ were the major degradation reactions of SM2 [30,31]. As shown in Figure 5a, the degradation of SM2 in Bi/mZVI+CA+NaCA system might be mainly through the hydroxylation and extrusion of SO_2_. In the pathway I, SM2 was hydroxylated by •OH to form P1 (or P2), then produced P5 and P9 (or P4 and P10) by cleavage of S-N bond, respectively. P9 further released SO_2_ to generate P8, and P10 also released SO_2_ to generate P11. On the other hand, in the pathway II, compound P3 was observed, which was most likely a product of SO_2_ extrusion. Subsequently, the •OH and •O_2_^−^ attacked the C-N bond, resulting in the generation of P6 and P7 (or P4 and P8). Eventually, all molecules were oxidized to CO_2_, H_2_O, NO_3_^−^, NH_4_^+^, and other small molecules under oxidation process [32]. 

Additionally, as shown in Figure 5b, there were three important and main processes in SMX degradation including hydroxylation, extrusion of SO_2_, and N-O bond breaking. Hydroxyl radicals can easily be added or substituted with aromatic groups [33,34]. Thus, in the pathway I, SMX was hydroxylated by •OH to form P13, then produced P16 and P10 by cleavage of S-N bond. P16 was further oxidized to P17 by breaking the N-O ring, and P10 was further oxidized to generate P11. In the pathway II, P14 obtained by SO_2_ extrusion was likely to further react to form P8 and P19, then these molecules were oxidized to some small molecules under ROS attack. Pathway III was mainly the process of the breaking of N-O ring, which was the main pathway of SMX degradation. •OH and •O_2_^−^ attacking on N-O ring resulted in the generation of P12 that could further generate P15 through hydroxyl addition, subsequently, the S-N bond was also attacked to form P10 and P17. The generated P10 was further oxidized to produce P11 with *m*/*z* = 110. Finally, all molecules were oxidized to various small molecules in Bi/mZVI+CA+NaCA system.

Generally, SD was mainly oxidized through some ways including hydroxylation, high electron cloud (such as N-S group, C-N group) bond cleavage, SO_2_ extrusion, and so on [35]. We detected seven by-products of SD in mass spectrometry (Figure S6) and found that the degradation pathways of SD were similar to SM2, mainly through hydroxylation and SO_2_ extrusion. As shown in Figure 5c, in the pathway I, the P18 obtained through hydroxyl addition in Bi/mZVI+CA+NaCA system. Then, due to the attack of •OH and •O_2_^−^, it further formed the production of P10 (*m*/*z* = 174) and P19 (*m*/*z* = 96) by S-N bond breaking. In the pathwayII, SD was extruded by SO_2_ to generate P20. Then, P8 and P19 were generated through the breaking of C-N bond on P20. Finally, these molecules were oxidized by ROS to generate small molecules and other substances (such as CO_2_, H_2_O, and NO_3_^−^). 

The core structure of SAs is p-aminobenzene sulfonamide, which has a benzene ring in its structure. The amino group on the sulfonamide is attached to a substituent group. SM2 and SD are a class of antibiotics substituted by six-membered heterocycles, but SMX contains a five-membered oxazole ring, and the N in the oxazole ring is the electron donor group. It indicates that the degradation pathway of SMX is probably different from that of SM2 and SD. The analysis of degradation intermediates confirmed this point. Based on the analysis of intermediate by-products of SAs produced in Bi/mZVI+CA+NaCA system, the proposed degradation pathways of SM2, SMX, and SD were similar, such as hydroxylation and the extrusion of SO_2_. In addition, based on the possible degradation pathways proposed by the LC-MS analysis results, we found that these three antibiotics all produced the same intermediate products (P8 or P10) at *m*/*z* = 110. However, SMX has an additional degradation pathway, which is reflected in the opening of the N-O bond on the ring, and this is probably the most important pathway for SMX degradation, since the five-membered ring on SMX is more vulnerable to hydroxyl attack and then opens. To some extent, SMX may have a better degradation effect in Bi/mZVI+CA+NaCA system than SM2 or SD, due to the addition of five-membered loop opening pathway of SMX. That is why the removal of SMX achieved the best degradation efficiency (74%) as mentioned above (Figure 3d), which also further proved our conjecture. 

## 3. Materials and Methods

### 3.1. Chemicals

Ferrous sulfate, bismuth nitrate, sodium borohydride, anhydrous ethanol, citric acid (CA), sodium citrate (NaCA), glacial acetic acid, methanol, acetonitrile, ferric nitrate, sulfamethazine (SM2), sulfamethoxazole (SMX), and sulfadiazine (SD) were supplied by Sinopharm Chemical Reagents Co., Ltd. (Shanghai, China). The 5-dimethyl-1-pyrroline N-oxide (DMPO) was purchased from Aladdin Industrial Company (Shanghai, China). Methanol and acetonitrile were HPLC grade, while other chemical reagents were analytical grade. All aqueous solutions were prepared using ultrapure water.

### 3.2. Particle Preparation

mZVI was purchased from Dingxin Chemical (Suzhou, China) with a diameter of about 100 mesh (147 μm). Bi/mZVI was prepared by sodium borohydride reduction method. Typically, 1 g mZVI and 0.03465 g Bi(NO_3_)_3_·5H_2_O were added into 100 mL denitrification ultrapure water. Nitrogen(N_2_) was continuously injected into the mixed solution for a period. 0.048 g NaBH_4_ was dissolved in 5 mL ultrapure water and was slowly dropped into the mixed solution for vacuum filtration. The collected solid material was washed several times with anhydrous ethanol, then centrifuged and vacuum-dried for use.

### 3.3. Characterization Analysis

The morphologies were recorded by scanning electron microscopy (SEM, Thermo Fisher Scientific, Breda, The Netherlands). The structures of samples were analyzed with X-ray diffraction (XRD, PANalytical B.V., EA Almelo, The Netherlands) under Cu Kα radiation (wavelength 0.154 nm). The chemical compositions were tested by X-ray photoelectron spectroscopy (XPS, Perkin-Elmer PHI Co., Waltham, MA, USA).

### 3.4. Degradation Procedure

Batch degradation experiments of Sulfonamides antibiotics were carried out in 60 mL sealed brown bottle. The bottles contained 1 g/L Bi/mZVI [29], and the concentration of CA and NaCA were 1 mM each in the whole reaction system. Typically, Bi/mZVI, CA, and NaCA were added to SM2 (0.2 mM), SMX (0.2 mM), SD (0.1 mM) solution, and the mixed solution of the three pollutants, respectively. The bottles were sealed with butyl-rubber stoppers and aluminum crimp caps, then incubated on a rotary shaker at room temperature in the dark. The whole reaction process had no pH adjustment. Solutions were taken at regular intervals, followed by immediate measurement of DO and subsequent chemical analyses. To further evaluate the stability of Bi/mZVI, the reacted Bi/mZVI was held by a permanent magnet, and then drained the supernatant. The same amount of CA, NaCA, and contaminants were immediately added to the separated sample to start a new cycle.

### 3.5. Analytic Methods

Samples were collected at given time intervals using a 1 mL syringe and then immediately filtered through a 0.22 μm syringe filter. High performance liquid chromatography (HPLC, Agilent 1100 chromatograph, Santa Clara, CA, USA) was used to quantitative analysis of organics equipped with a C-18 column (150 mm × 4.6 mm). The mobile phase of SM2 (20:80 *v/v*), SMX (30:70 *v/v*), and SD (20:80 *v/v*) were acetonitrile and 0.1% acetic acid. The flow rate of the mobile phase was 0.8 mL/min, and the detection wavelengths were 260 nm, 260 nm, and 247 nm, respectively.

The intermediate products of SAs (SM2, SMX, and SD in the mixed liquid) were analyzed using a high-resolution Q Exactive tandem MS system coupled to an UPLC system (HR-LC-MS, Thermo Fisher Scientific, Waltham, MA, USA) equipped with a Hypersil GOLD Column (100 mm × 2.1 mm). The mobile phase was acetonitrile and water (both contain 0.1% acetic acid), and gradient elution was adopted. The flow rate of the mobile phase was 0.25 mL·min^−1^ and the detection wavelength was 270 nm. The pH and DO value were measured by MP525 pH/DO meter (Shanghai San-Xin Instrumentation, Shanghai, China) and COD was measured by DR1010 COD meter (Shanghai Shilu Instrumentation).

## 4. Conclusions

In this work, we used the bismuth modified commercial zero-valent iron to oxidation of SAs in citrate buffer solution. The generated •OH was involved in the degradation process. At this process, the bismuth acts as a catalyst to reduce the activation energy of the system to further produce H_2_O_2_ from O_2_. Additionally, the complexation reaction between iron and citric ion occurred to further reduce H_2_O_2_ to form •OH. The COD removal of the mixed high concentration antibiotic solution even achieved 39% within 3h. The analysis of LC-MS results revealed that SM2, SMX, and SD in the mixed solution can degrade pollutants by the ways of hydroxylation and SO_2_ extrusion. Furthermore, the three antibiotics produced the same intermediate products (P1 or P10) with *m*/*z* = 110, due to the presence of p-aminobenzene sulfonamide. The results indicated that the process can degrade SAs that have similar structures. Therefore, the Bi/mZVI+CA+NaCA system was facile, environmentally friendly, and inexpensive, and could be potentially scaled-up.

## Data Availability

The study did not report any data.

## Supplementary Materials

The following supporting information can be downloaded at: https://www.mdpi.com/article/10.3390/antibiotics11010051/s1, Figure S1: Degradation efficiency of SM2 by mZVI doped with different amount of Bi; Figure S2: Plots of -ln(C/C_0_) versus time for the (a) SM2 (b) SMX (c) SD degradation in different systems; (d) Plots of -ln(C/C_0_) versus time for the mixed liquid in Bi/mZVI+CA+NaCA system; Figure S3: Three cycles of degradation of (a) SM2 (b) SMX (c) SD Bi/mZVI+CA+NaCA system; Figure S4: The pH change trend of (a) SM2 (b) SMX (c) SD in different systems; (d) The pH change trend of mixed pollutant in Bi/mZVI+CA+NaCA system; Figure S5: Total ion chromatogram of mixed liquid at 0 min, 10 min, and 60 min; Figure S6: Mass spectrometry of intermediates in ESI positive ions during the degradation of SAs in Bi/mZVI+CA+NaCA system; Table S1: List of reaction rate constants of SAs; Table S2: The structural information of the possible intermediates products.
