# Supplementary material for: Bi/mZVI Combined with Citric Acid and Sodium Citrate to Mineralize Multiple Sulfa Antibiotics: Performance and Mechanism
Source: Antibiotics (Basel). 2022 Jan 1;11(1):51. doi: 10.3390/antibiotics11010051 (PMC8773326; doi:10.3390/antibiotics11010051)
Supplement: Supplementary file 1 [file antibiotics-11-00051-s001.zip › antibiotics-1492982-supplementary.pdf]

Supporting Materials

Bi/mZVI combined with citric acid and sodium citrate to mineralize multiple sulfa antibiotics: Performance and mechanism

Xiaoming Su [a], Hao Lv [a], Jianyu Gong [a,*] and Man Zhou [b,*]

[a] School of Environmental Science and Engineering, Huazhong University of Science and Technology, Wuhan, 430074, China

[b] Hubei Electromechanical Research Institute Co Ltd.,Wuhan, 430070, China

* Correspondence: jygong@hust.edu.cn (J.G.); mfkjlc@163.com (M.Z.)

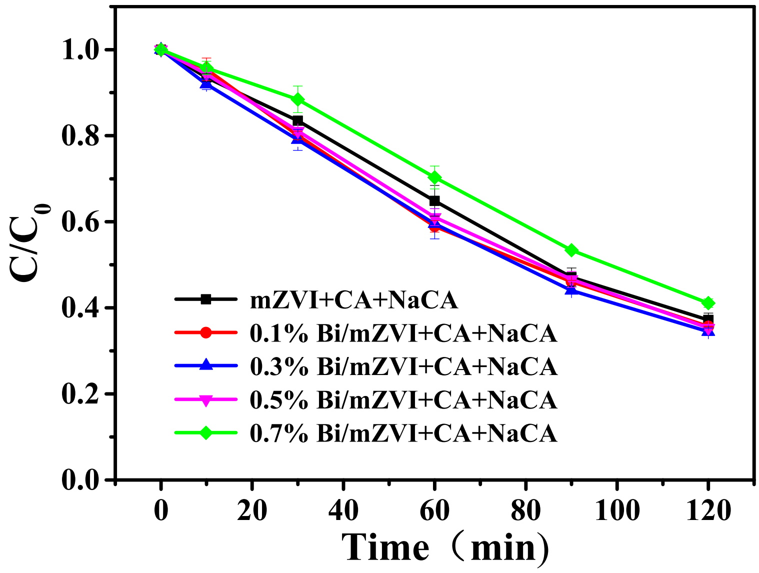

Figure S1. Degradation efficiency of SM2 by mZVI doped with different amount of Bi.

The kinetics study of SAs degradation was carried out (Figure S2). The reaction rate constants are shown in table S1 and the degradation of SAs follows a quasi-first order kinetic process. For the degradation of single SM2 (Figure S2a), SMX (Figure S2b) and SD (Figure S2c), the reaction rate constants of Bi/mZVI+CA+NaCA system are higher than those of mZVI, Bi/mZVI and mZVI+CA+NaCA system. The results showed that Bi/mZVI+CA+NaCA system had obvious advantages in the degradation of sulfa antibiotics. Then, we investigated the kinetics process of the mixed liquid in Bi/mZVI+CA+NaCA system, and found that the reaction rate constants of SM2, SMX and SD were 6.7×10^{-3}, 1.1×10^{-2} and 7.5×10^{-3}, respectively (Figure S2d). These results indicate that CA and NaCA played an important role in the degradation of SAs.

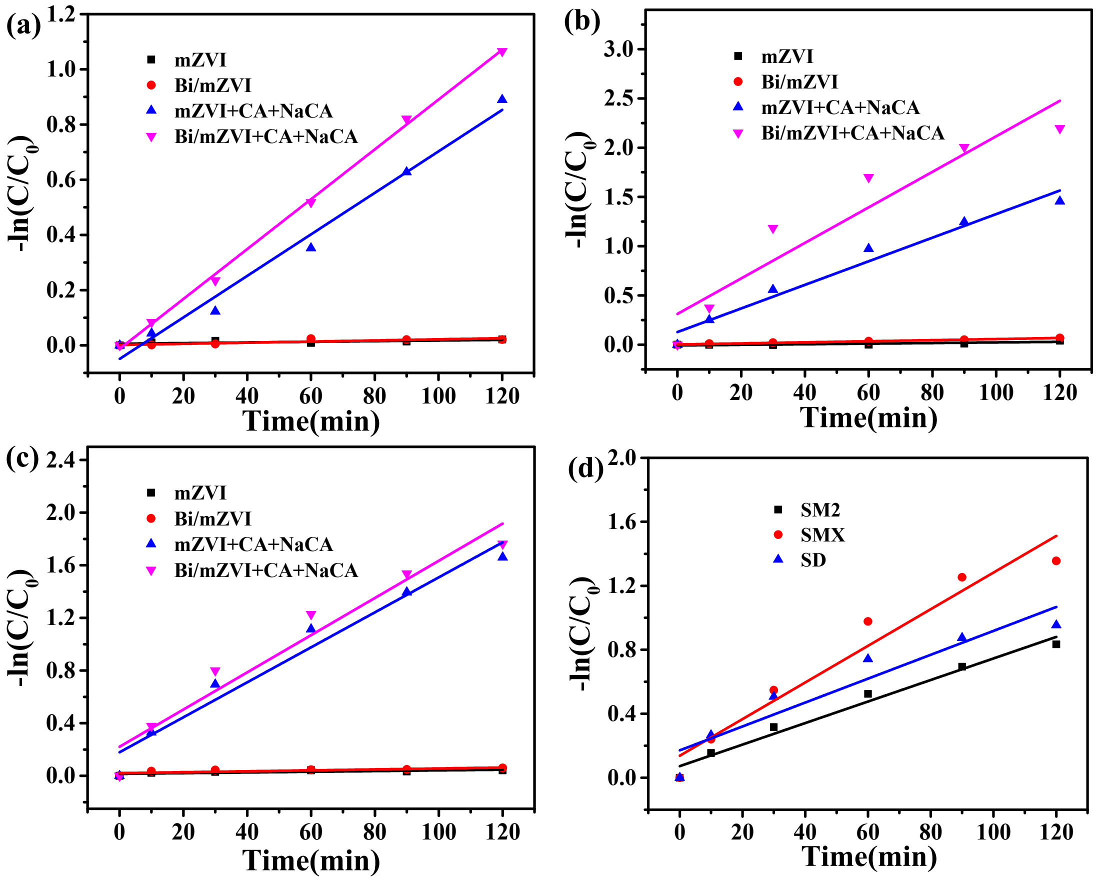

Figure S2. Plots of -ln(C/C$_0$) versus time for the (**a**) SM2 (**b**) SMX (**c**) SD degradation in different systems; (**d**) Plots of -ln(C/C$_0$) versus time for the mixed liquid in Bi/mZVI+CA+NaCA system.

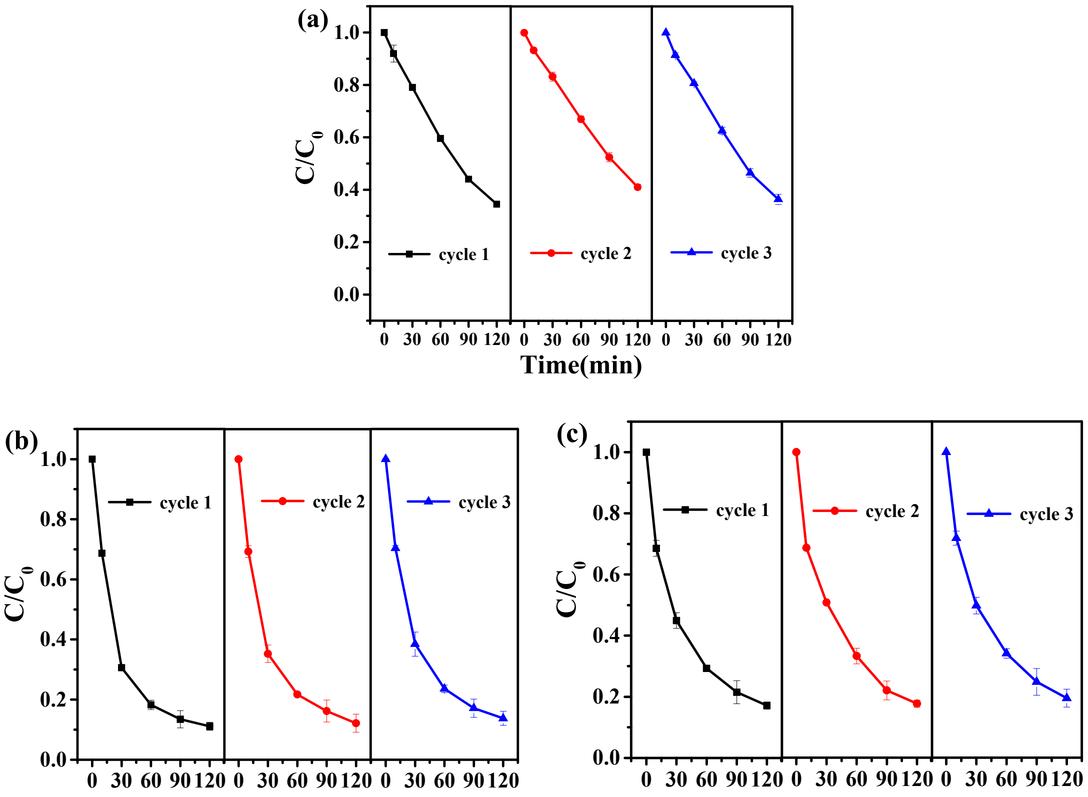

Figure S3. Three cycles of degradation of (**a**) SM2 (**b**) SMX (**c**) SD Bi/mZVI+CA+NaCA system.

Table S1. List of reaction rate constants of SAs.

component	SAs	system	The Reaction Rate Constant
A single component	SM2	mZVI	1.2×10^{-4}
		Bi/mZVI	2.1×10^{-4}
		mZVI+CA+NaCA	7.5×10^{-3}
		Bi/mZVI+CA+NaCA	9×10^{-3}
	SMX	mZVI	3.1×10^{-4}
		Bi/mZVI	5.5×10^{-4}
		mZVI+CA+NaCA	1.1×10^{-2}
		Bi/mZVI+CA+NaCA	1.8×10^{-2}
	SD	mZVI	2.6×10^{-4}
		Bi/mZVI	3.6×10^{-4}
		mZVI+CA+NaCA	1.3×10^{-2}
		Bi/mZVI+CA+NaCA	1.4×10^{-4}
Hybrid components	SM2	Bi/mZVI+CA+NaCA	6.7×10^{-3}
	SMX		1.1×10^{-2}
	SD		7.5×10^{-3}

The changes of pH of SAs in different systems during the reaction were discussed. As shown in Figure S4a and Figure S4c, the presence of CA and NaCA resulted in a lower initial PH of the solution, and the pH increased gradually during the degradation reaction in all systems. As shown in Figure S4b, the presence of CA and NaCA also resulted in a lower initial pH value, and the PH value in all systems after 2 h reaction was about 6.3. In addition, the pH change of mixed liquid in Bi/mZVI+CA+NaCA system was tracked (Figure S4d). The pH value was gradually increasing to 6.31 from 4.72. The increased pH trends for all cases resulted from the release of Fe^{2+} or Fe^{3+} when zero-valent iron degraded pollutants.

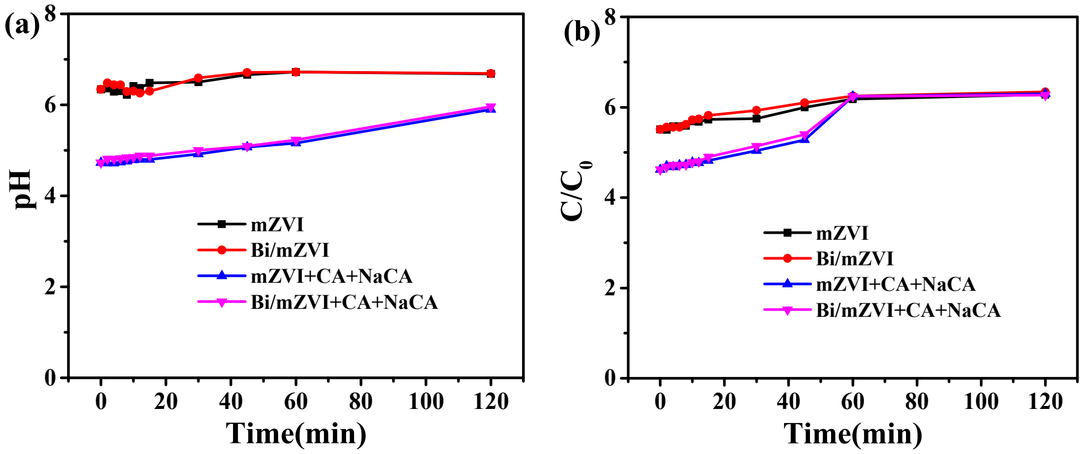

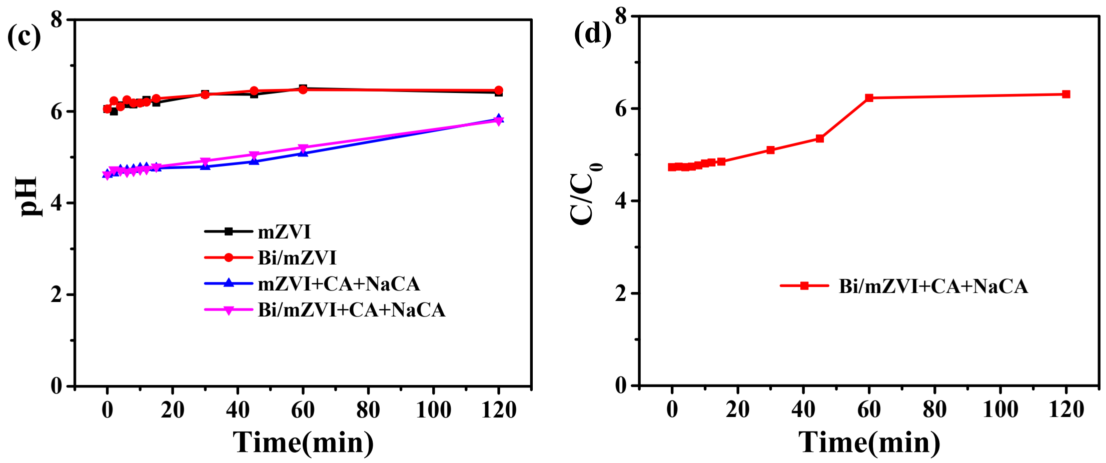

Figure S4. The pH change trend of (a) SM2 (b) SMX (c) SD in different systems; (d) The pH change trend of mixed pollutant in Bi/mZVI+CA+NaCA system.

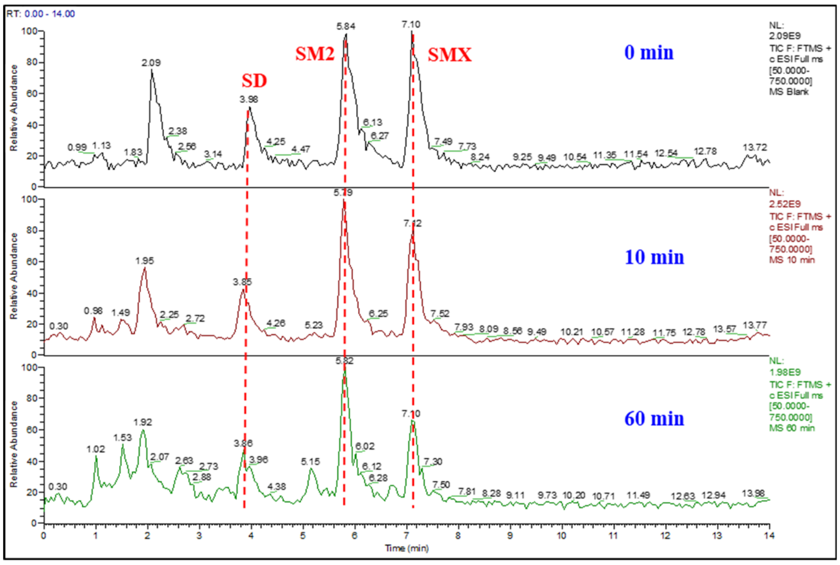

Figure S5. Total ion chromatogram of mixed liquid at 0 min, 10 min and 60 min.

Figure S6 showed the mass spectrometry of intermediates in ESI positive ions during the degradation of SAs in Bi/mZVI+CA+NaCA system. The contaminants of SM2, SMX and SD detected in the mixed liquid at 0 min was shown in figure S6 a. According to the m/z value of the compound, the chemical formula of the compound can be obtained, so as to predict what kind of substance the compound is. Figure S6 b–q shows 20 intermediates detected by HR-LC-MS in the process of degradation reaction. Then, according to the chemical structure formula of the degraded substrate, the possible chemical structural formula of these 20 compounds was further inferred. Finally, the possible degradation pathway of pollutants was proposed by the chemical structure formula obtained.

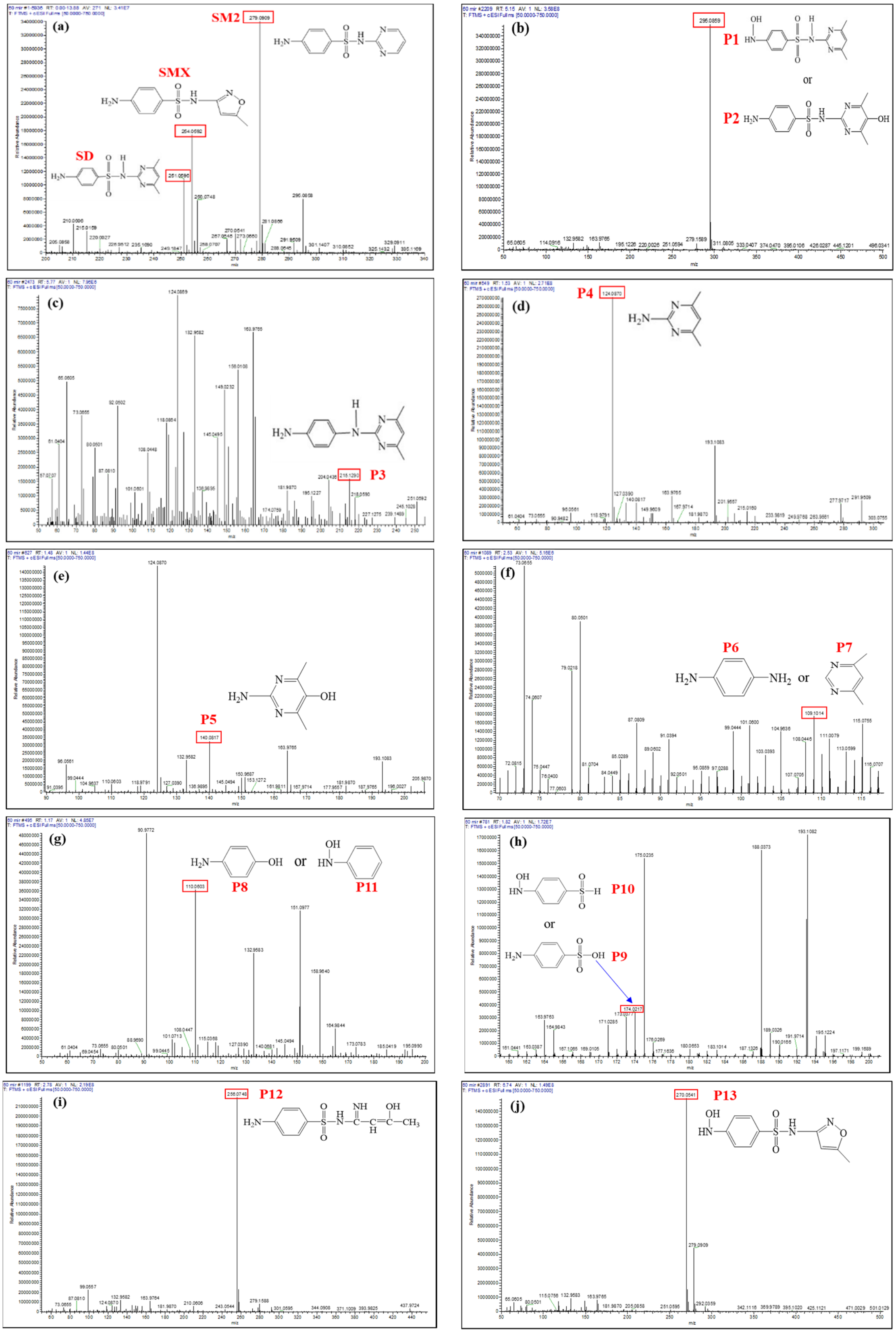
(a)
SM2
279.0909
SMX
254.0592
SD
251.0690
(b)
296.0859
P1
or
P2
(c)
215.1290
P3
(d)
P4
124.0870
(e)
P5
140.0817
(f)
P6
P7
or
109.1014
(g)
110.0603
P8
or
P11
(h)
P10
or
P9
174.0217
(i)
256.0748
P12
(j)
270.0541
P13
Relative Abundance
m/z

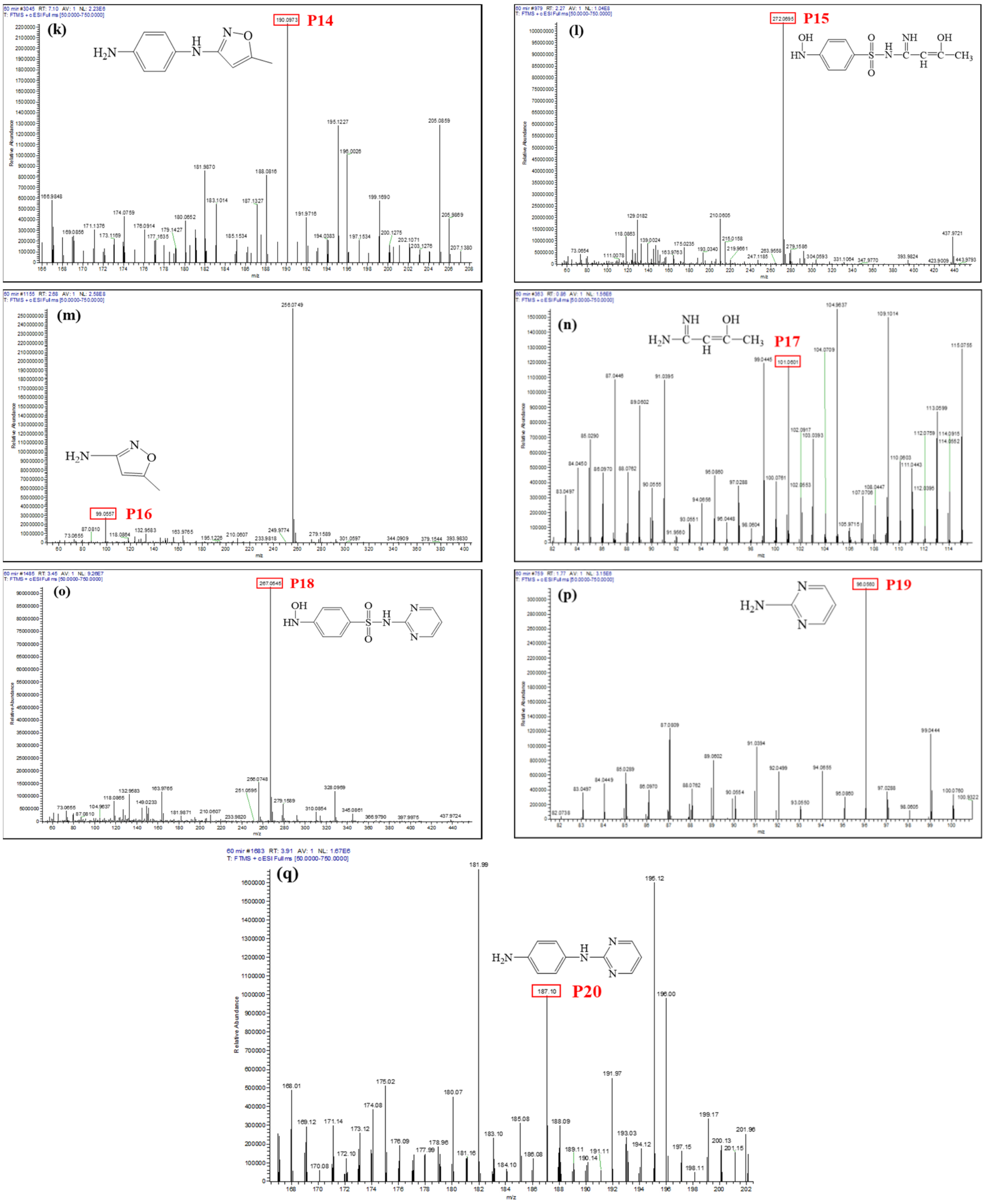

Figure S6. Mass spectrometry of intermediates in ESI positive ions during the degradation of SAs in Bi/mZVI+CA+NaCA system.

Table S2. The structural information of the possible intermediates products.

Compounds	Formula	m/z	Proposed structure
SM2	$C_{12}H_{14}N_4O_2S$	279	
SMX	$C_{10}H_{11}N_3O_3S$	254	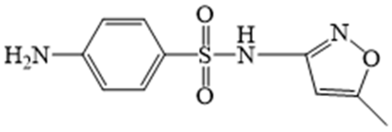

**SD**	**$C_{10}H_{10}N_4O_2S$**	**251**	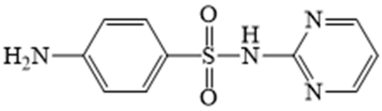
**P1**	**$C_{12}H_{14}N_4O_3S$**	**295**	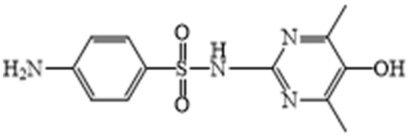
**P2**	**$C_{12}H_{14}N_4O_3S$**	**295**	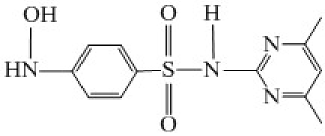
**P3**	**$C_{12}H_{14}N_4$**	**215**	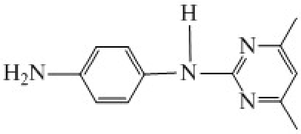
**P4**	**$C_6H_9N_3$**	**124**	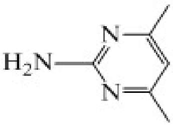
**P5**	**$C_6H_9N_3O$**	**140**	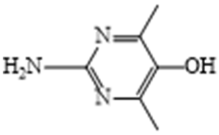
**P6**	**$C_6H_8N_2$**	**109**	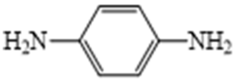
**P7**	**$C_6H_8N_2$**	**109**	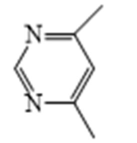
**P8**	**C_6H_7NO**	**110**	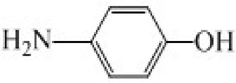
**P9**	**$C_6H_7NO_3S$**	**174**	
**P10**	**$C_6H_7NO_3S$**	**174**	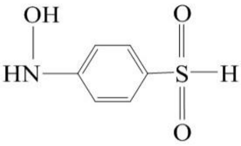
**P11**	**C_6H_7NO**	**110**	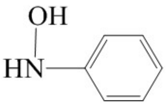
**P12**	**$C_{10}H_{13}O_3N_3S$**	**256**	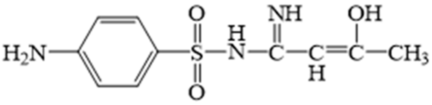
**P13**	**$C_{10}H_{11}N_3O_4S$**	**270**	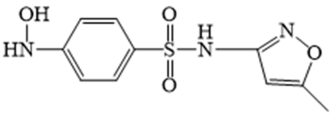

**P14**	**$C_{10}H_{11}N_3O$**	**190**	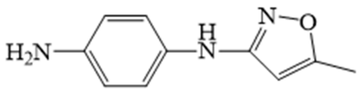
**P15**	**$C_{10}H_{13}O_4N_3S$**	**272**	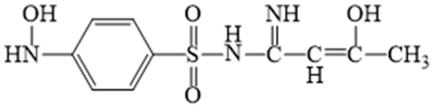
**P16**	**$C_4H_6N_2O$**	**99**	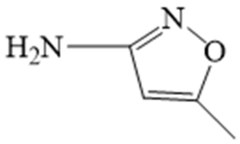
**P17**	**$C_4H_8N_2O$**	**101**	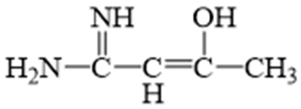
**P18**	**$C_{10}H_{10}N_4O_3S$**	**267**	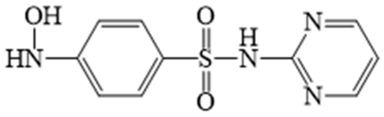
**P19**	**$C_4H_5N_3$**	**96**	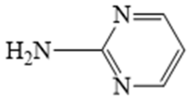
**P20**	**$C_{10}H_{10}N_4$**	**187**	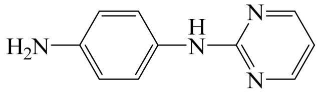
